# Influence of heat generation/absorption and stagnation point on polystyrene–TiO_2_/H_2_O hybrid nanofluid flow

**DOI:** 10.1038/s41598-021-01747-9

**Published:** 2021-11-17

**Authors:** Sadaf Masood, Muhammad Farooq, Aisha Anjum

**Affiliations:** 1 Department of Mathematics, Riphah International University, Islamabad, 44000 Pakistan; 2 Department of Mathematics and Statistics, University of Haripur, Haripur, Pakistan; 3 Department of Mathematics, NUML, Islamabad, Islamabad Pakistan

**Keywords:** Engineering, Mathematics and computing, Nanoscience and technology

## Abstract

This article focuses on hybrid nanofluid flow induced by stretched surface. The present context covers stagnation point flow of a hybrid nanofluid with the effect of heat generation/absorption. Currently most famous class of nanofluids is Hybrid nanofluid. It contains polystyrene and titanium oxide as a nanoparticles and water as a base fluid. First time attributes of heat transfer are evaluated by utilizing polystyrene–TiO_2_/H_2_O hybrid nanofluid with heat generation/absorption. Partial differential equations are converted into ordinary differential equation by using appropriate transformations for heat and velocity. Homotopy analysis method is operated for solution of ordinary differential equations. Flow and heat are disclosed graphically for unlike parameters. Resistive force and heat transfer rate is deliberated mathematically and graphically. It is deduced that velocity field enhanced for velocity ratio parameter whereas temperature field grows for heat generation/absorption coefficient. To judge the production of any engineering system entropy generation is also calculated. It is noticed that entropy generation grows for Prandtl number and Eckert number while it shows opposite behavior for temperature difference parameter.

## Introduction

Heat transmission plays a vital role in many respects for instance in refrigeration, power generation, thermoelectric devices, heat exchangers, roofing materials, food processing, radiative cooling and thermal energy storage etc. Therefore it is advantageous to enhance the production of heat transfer machines adopted in these areas. Thermal conductivity is the crucial framework in heat transfer problems. Ethylene glycol, water and oils have low thermal conductivity. Nanomaterials like oxides of metals, carbides etc. are included in the host fluid for intensification of thermal conductivity. Choi^1^ instigated about nanofluids. Hybrid nanofluids seeks the intention of researchers and scientists currently. It consists of two or more non-identical particles having size less than 100 nm. Here, we take polystyrene and titanium oxide as nanoparticles due to their wide use in pharmaceuticals, automotive industry, IT equipments (TV, Computers, laptops), food packing industry, construction, household industry, cosmetics, fabrics and textiles. Waini et al.^2^ avails bvp4c to conclude the heat transfer of hybrid nanofluid with shear flow. They checked the stability of a solutions and concluded that one solution is stable from the dual solutions. Influence of CNT—Fe_3_O_4_/H_2_O hybrid nanofluid on infinite rotating disk was studied by Tassaddiq et al.^3^. They observed that heat transfer of CNT—Fe_3_O_4_/H_2_O hybrid nanofluid is greater as compared with Fe_3_O_4_/H_2_O. Tayebi et al.^4^ analyzed the attributes of hybrid nanofluid (containing Cu and Al_2_O_3_ nanoparticles) bounded by elliptical cylinders with natural convection. He investigated that entropy generation grows for higher Rayleigh number. Peristaltic flow of hybrid nanofluid with entropy generation was executed by Zahid et al.^5^. Shooting method is utilized to perceive numerical solutions. It is noticed that enhanced Hall parameter decays the heat transfer rate as well as entropy generation. Yusuf et al.^6^ takes stretching sheet with nonlinear radiations on hybrid nanofluid and investigated that entropy generation rate rises for radiation parameter. Wanatasanapan et al.^7^ takes temperature range 30–70 °C and fixed volume fraction 1.0% of TiO_2_ and Al_2_O_3_ particles. They concluded that hybrid nanofluid with 50:50 ratio at 70 °C has maximum thermal conductivity. Matlab was used by Said et al.^8^ for hybrid nanofluid at distinct concentrations. Enhanced nanoparticle volume concentration grows the entropy generation. 19.14% thermal conductivity enhancement was noticed at 60 °C. Abbas et al.^9^ considered a hybrid nanofluid in a moving cylinder with slip and inclined MHD. Consequences of silver-CuO/H_2_O nanofluid with stagnation point and stretching sheet are discussed by Arani et al.^10^. They used R–K method with shooting technique. They conclude that heat transfer rate grows 100% for hybrid nanofluid with suction/injection parameter. Enhancement of heat transfer in a car radiator with hybrid nanofluid was studied by Li et al.^11^. He found that at 0.4% volume fraction 32.01% thermal conductivity rises. Waini et al.^12,13^ disclosed the impact of surface heat flux and stagnation point on a stretching/shrinking cylinder filled with a hybrid nanofluid. They also studied about squeezed flow over a permeable sensor surface. Results showed that unique solution was obtained for $\lambda \ge - 1$. They also conclude that the first solution is stable only. Different researchers take polystyrene and titanium oxide nano particles and perform numerous experiments^14–18^.

Excessive applications of stagnation point flow of Newtonian as well as Non-Newtonian fluid seeks the researchers intention. A point where fluid is static by the object is called stagnation point. It is classified as oblique and orthogonal stagnation point. Stagnation point undergoes a highest pressure and highest heat transmission. Innumerable applications of stagnation point flows in engineering, home industry, aerodynamic industry and in metallurgy are noticed. They include cooling of plates, nuclear reactor cooling, tinning of wires and wire drawing etc. Naganthran et al.^19^ described the flow of viscoelastic fluid past a shrinking sheet with oblique stagnation point. They concluded that enhanced mass flux parameter strengthens the heat transfer rate. Consequences of chemical reaction on CNTs along with stagnation point was founded by Khan et al.^20^. Magnified velocity ratio parameter decays the drag force. Ascendancy of Maxwell fluid with suction/injection was illustrated by Ahmed et al.^21^. Porous rotating disk was also considered. Numerical results showed that rotation parameter enhanced the Nusselt number at the surface. Moshkin et al.^22^ elaborated the flow of unsteady Maxwell fluid by transforming the equations into the Lagrangian coordibates. Weidman^23^ contemplated a flow along a rotating plate and revealed the influence of Hiemenz stagnation point on this plate. He gave good comparision with Hannah's consideration. The out turn of Homann stagnation point on a non-Newtonian fluid regarding a stable plate was initiated by Mahapatra et al.^24^. They deduced that viscoelastic parameter grows the velocity profile. Azhar et al.^25^ studied about heat generation and viscous dissipation of Jeffrey fluid along with stagnation point. Stretching ratio parameter enhanced the drag force as well as Sherwood number. Flow of MHD Carreau fluid induced by stretching surface was instigated by Chu et al.^26^. He examined flow about stagnation point. Both heat and mass will transfer more fastly by increasing velocity ratio parameter. Shah et al.^27^ takes a Riga plate and found the out turns of stagnation point and mixed convection along with porous medium. Darcy number decays the velocity field while grows the skin friction. Awan et al.^28^ assumed MHD second grade fluid with oblique stagnation point. The flow was induced by oscillatory surface. He showed that Sherwood number and heat transfer rate boosts up for larger suction parameter. Effect of stagnation point on Cu–Al_2_O_3_/water hybrid nanofluid with shrinking cylinder was investigated by Waini et al.^29^. The findings revealed that velocity grows for rising Reynold number. Influence of mixed convection flow on hybrid nanofluid flow with stagnation point was originated by Zainal et al.^30^. They concluded that heat transfer rate and skin friction coefficient boosts up for growing suction parameter.

The fluid flows initiated by stretching surfaces have significant preferences in engineering as well as industrial applications. For example, cooling of stripes, glass fiber, extrusion processes, paper production, drawing of paper film, crystal growth and production of rubber sheets etc. To obtain output of acceptable quality stretching and cooling of sheets played an important role. In past, many researchers investigated the flow induced by stretching sheets. Buoyancy effects on hybrid nanofluid flow over an exponentially stretching sheet with stagnation point was scrutinized by Waini et al.^31^. They considered Al_2_O_3_–Cu/Water hybrid nanofluid and bvp4c Matlab solver for finding the solution of the problem. They noticed that first solution of heat transfer coefficient increased for greater mixed convection parameter. Khashi’ie et al.^32^ examined the dual solutions of hybrid nanofluid with prescribed heat flux. They assumed cylinder as well as flat plate and found the results. Bvp4c Matlab solver was employed. They obtained that concentration of Alumina particles $\Phi_{1} = 0.5\%$ and concentration of copper particles $\Phi_{2} = 1.5\%$ has extortionate heat transfer rate. Similarly Zainal et al.^33–35^ considered hybrid nanofluid with viscous dissipation, stagnation point and velocity slip effects. They also take exponentially stretching sheet with quadratic velocity and gained different remarkable results.

The crucial element in industrial, geographical and engineering processes is transmission of heat. They include numerous applications such as thermal insulation, geothermal supplies, cooling of electrical devices, metal casting etc. The temperature difference within a body is the key factor for heat generation/absorption. Two dimensional flow of Oldroyd-B fluid with heat generation/absorption under Cattaneo–Christov approach was developed by Ibrahim et al.^36^. They utilized finite element method for solution of the problem. The results revealed that heat transfer rate enhanced for increased heat generation/absorption parameter. Outcomes of hybrid nanofluid over exponentially stretching sheet with heat generation was examined by Zainal et al.^37^. Suction parameter strengthens the rate of heat transfer. Hafeez et al.^38^ considered a rotating disk having Oldroyd-B fluid with the influence of heat generation/absorption. They concluded that the temperature is an increasing function for magnetic number whereas it decays for thermal relaxation time parameter. Consequences of heat generation/absorption on hybrid nanofluid in a circular annulus was founded by Tayabi et al.^39^. They take Cu and Al_2_O_3_ as nanoparticles. They found that the influence of IHG/A modifies the heat exchange rate in the annulus.

Conception of entropy in thermodynamic system was prescribed by Rudolf Clausius in 1850s. It is the amount of thermal energy per unit temperature which is unattainable for performing beneficial tasks. The quantity of entropy assembled in irreversible processes is termed as entropy production. For example heat exchange, heat engines, fluid flows, heat pumps, power plants, air conditioners and refrigeration etc. It determines the execution of thermodynamical system. At first Bejan^40^ studied about entropy generation. He explained the significant steps of entropy depreciation. Gholamalipour et al.^41^ studied the entropy generation of nanofluid in a permeable annulus. For lesser Darcy and Rayleigh number greater disturbance in entropy production is noticeable. Dutta et al.^42^ considered a rhombic shape closed pattern pervaded by Cu–water nanofluid and investigates about entropy generation. He showed that increment in Ha decays the entropy production rate. Khan et al.^43^ discussed the impact of joule heating on Casson fluid passing through a revolving cylinder. Entropy generation shows increasing trend for larger Brinkman number. Attributes of entropy production in Newtonian fluid with Darcy model was analyzed by Ambreen et al.^44^. Cho^45^ takes a square cavity whose some walls are heated and filled it by Cu–water nanofluid. Along this he considered a porous medium inside the cavity and then measure the entropy production rate. For a fixed Rayleigh number, entropy rate enhanced with enlarged Darcy number. Influence of natural convection in elliptical cavity pervaded by hybrid nanofluid was inspected by Tayebi et al.^4^. Zahid et al.^5^ explained that low entropy production occurs for higher Hall parameter. Li et al.^46^ examined the thermal radiation effect in a tilted square cavity. He also analyzed the entropy production rate here and found that Rayleigh number grows the entropy production rate. Sachica et al.^47^ scrutinized the Al_2_O_3_–water nanofluid in a rectangular channel and numerically investigate it. Nano particle volume fraction decreases the entropy generation rate.

Extraordinary enhancement in thermal conductivity is noticed for hybrid nanofluids in comparision with ordinary nanofluids. Therefore have innumerable applications in home industry, automotive industry, engineering, for cancer treatment, cosmetics, pharmaceuticals, food pakaging, paper plastics, fabrics, ceramics, paints, food colorants and in soaps as well. Here the key objective is to discuss the characteristics of polystyrene–TiO_2_/H_2_O hybrid nanofluid flow with heat generation/absorption. Stagnation point is also contemplated in momentum equation. We take advantage of congrous transformations for transmutation of partial differential equations into non dimensionalized ordinary differential equations. Homotopic methodology^48–54^ is executed for series solution. Ramification of incompatible parameters are interpreted graphically. Mathematical expression of drag force is calculated and Nusselt number is manifested graphically. Entropy generation rate is also exposed through graphs.

## Formulation

Analysis of two dimensional hybrid nanofluid suppressed with polystyrene and titanium oxide (TiO_2_) particles has carried out. Influence of stagnation point on flow pattern is also discussed. Impact of heat generation/absorption is also figure out. We take stretching velocity $u = U_{w} \left( x \right) = ax$ at $y = 0$. Free stream velocity $u = U_{e} \left( x \right) = bx$ is considered at $y \to \infty$. Persistent temperature is presumed at both plate surface and ambient fluid.

After implementation of boundary layer approximation the ruling equations appears as^55^:

1 $$\frac{\partial u}{{\partial x}} + \frac{\partial v}{{\partial y}} = 0,$$

2 $$u\frac{\partial u}{{\partial x}} + v\frac{\partial u}{{\partial y}} = U_{e} \frac{{dU_{e} }}{dx} + \upsilon_{hnf} \frac{{\partial^{2} u}}{{\partial y^{2} }},$$

3 $$u\frac{\partial T}{{\partial x}} + v\frac{\partial T}{{\partial y}} = \alpha_{hnf} \frac{{\partial^{2} T}}{{\partial y^{2} }} +({{T-T_{\infty}}}) \frac{Q}{{\left( {\rho C_{p} } \right)_{hnf} }},$$

here $u$ and $v$ are symbolized as velocity constituents $x$ and $y$ respectively. $U_{e}$ designated as free stream velocity, $\upsilon_{hnf}$ stands for kinematic viscosity of hybrid nanofluid, $\rho_{hnf}$ characterizes density of hybrid nanofluid, heat capacity of hybrid nanofluid is denoted by $\left( {\rho C_{p} } \right)_{hnf}$, $\alpha_{hnf}$ indicates thermal diffusivity of hybrid nanofluid. $T$ stands for fluid temperature.

Corresponding boundary conditions are

4 $$u = U_{w} \left( x \right) = ax,v = 0,T = T_{0} + dx^{2} ,\;{\text{at}}\;y = 0,$$

5 $$u = U_{e} \left( x \right) = bx{,}T \to T_{\infty } ,\;{\text{at}}\;y \to \infty .$$

here $u$ and $v$ denotes velocity components, $U_{w} (x)( = ax)$ stands for stretching velocity, $T_{0}$ denotes reference temperature, $T_{w}$ is the temperature of plate, ambient temperature is represented by $T_{\infty }$.

Exploiting the transformations^56^

6 $$u = axf^{\prime}(\eta ),v = - \sqrt {a\nu } f(\eta ),\eta = \sqrt {\frac{a}{\nu }} y,\theta \left( \eta \right) = \frac{{T - T_{\infty } }}{{T_{w} - T_{\infty } }},$$

Equation (1) is fulfilled consistently. Though Eqs. (2–5) appears as:

7 $$f^{\prime\prime\prime} - B(1 - \Phi_{1} )^{2.5} (1 - \Phi_{2} )^{2.5} ((f^{\prime})^{2} - ff^{\prime\prime}) + A^{2} = 0,$$

8 $$\left( {\frac{{B_{1} }}{\Pr }\theta^{\prime\prime} + \lambda \theta } \right)\,\frac{1}{{D_{1} }} + f\theta^{\prime} - 2\theta f^{\prime} = 0,$$

9 $$f^{\prime}(0) = 1,\, \, f(0) \, = 0,\, \, f^{\prime}(\infty ) = A,$$

10 $$\theta (0) = 1,\, \, \theta (\infty ) = 0,\, \,$$

In these equations $A$ denotes velocity ratio parameter, $\Pr$ symbolizes the Prandtl number, $\lambda$ is heat generation/absorption parameter. Algorithmic representation of these quantities are specified as:

11 $$\begin{aligned} \, A = & \frac{b}{a} \, ,\, \, \mathop {\Pr }\limits = \frac{\upsilon }{\alpha },\, \, \lambda = \frac{Q}{{\left( {\rho C_{p} } \right)_{f} a}},\,B_{1} = \frac{{k_{hnf} }}{{k_{f} }},Ec = \frac{{u_{w}^{2} }}{{C_{p} (T_{w} - T_{\infty } )}}, \\ B = & \left[ {\left( {1 - \Phi_{2} } \right)\,\left\{ {\left( {1 - \Phi_{1} } \right) + \Phi_{1} \frac{{\rho_{s1} }}{{\rho_{f} }}} \right\} + \Phi_{2} \frac{{\rho_{s2} }}{{\rho_{f} }}} \right], \\ D_{1} = & \left[ {\left( {1 - \Phi_{2} } \right)\,\left\{ {\left( {1 - \Phi_{1} } \right) + \Phi_{1} \frac{{\left( {\rho C} \right)_{s1} }}{{\left( {\rho C} \right)_{f} }}} \right\} + \Phi_{2} \frac{{\left( {\rho C} \right)_{s2} }}{{\left( {\rho C} \right)_{f} }}} \right], \\ \end{aligned}$$

Mathematical form of drag force is prescribed as:

12 $$C_{f} = \frac{{\tau_{w} }}{{\rho U_{w}^{2} }},$$

Non dimensionalized configuration is expressed as

13 $$C_{f} Re_{x}^{1/2} = - \frac{1}{{(1 - \Phi_{1} )^{2.5} (1 - \Phi_{2} )^{2.5} }}f^{\prime\prime}(0),$$

Nusselt number is declared as

14 $$Nu = \frac{{xq_{w} }}{{k_{f} (T_{w} - T_{\infty } )}},$$

Its undimensional form is as below

15 $$NuRe_{x}^{1/2} = - \frac{{k_{hnf} }}{{k_{f} }}\theta^{\prime}(0),$$

here local Reynolds number is symbolized by $Re_{x} = U_{w} (x)x/\nu$.

## Entropy generation

Here our principal focus is to evaluate the irreversibilities of a system through entropy generation. Mathematically it is given as

16 $$E_{G} = \frac{{k_{hnf} }}{{(T_{\infty } )^{2} }}\left( {\frac{\partial T}{{\partial y}}} \right)^{2} + \frac{{\mu_{hnf} }}{{T_{\infty } }}\left( {\frac{\partial u}{{\partial y}}} \right)^{2} ,$$

Dimensionless numerical formula for entropy generation is demonstrated as

17 $$N_{s} = \frac{{(T_{\infty } )^{2} \left( {\tfrac{\eta }{y}} \right)^{2} }}{{k_{hnf} (T_{w} - T_{\infty } )^{2} }}E_{G} ,$$

Achieved non dimensional form is

18 $$N_{s} = \theta^{\prime 2} + \frac{Ec\;\Pr }{{\Omega (1 - \Phi_{1} )^{2.5} (1 - \Phi_{2} )^{2.5} }}\frac{{k_{f} }}{{k_{hnf} }}f^{\prime \prime 2} ,$$

Bejan number is expressed as

19 $$Be = \frac{{\text{Entropy generation due to thermal irreversibility}}}{{\text{Total entropy generation}}},$$

20 $$Be = \frac{{\theta^{\prime 2} }}{{\theta^{\prime 2} + \tfrac{Ec\Pr }{{\Omega (1 - \Phi_{1} )^{2.5} (1 - \Phi_{2} )^{2.5} }}\tfrac{{k_{f} }}{{k_{hnf} }}f^{\prime \prime 12} }},$$

## Solution methodology

### Homotopic solutions

Homotopic method was exposed by Liao^48^. This method is utilized for finding the series solutions of highly non-linear problems. It is independent of any small or large parameter. Convergence can be find and control easily. Initial guesses and linear approximations are free to choose. They are intimated as follows:

21 $$f_{0} \left( \eta \right) = A\eta - (A - 1)\left( {1 - \exp \left( { - \eta } \right)} \right),$$

22 $$\theta_{0} (\eta ) = exp( - \eta ).$$

23 $${\mathbf{L}}_{f} \left( f \right) = \frac{{d^{3} f}}{{d\eta^{3} }} + \frac{{d^{2} f}}{{d\eta^{2} }},\, \, {\mathbf{L}}_{\theta } \left( \theta \right) = \frac{{d^{2} \theta }}{{d\eta^{2} }} - \theta ,\, \,$$

with

24 $${\mathbf{L}}_{f} \left[ {\left( {C_{1} + C_{2} \eta } \right) + C_{3} \exp ( - \eta )} \right] = 0,$$

25 $${\mathbf{L}}_{\theta } \left[ {C_{4} \exp (\eta ) + C_{5} \exp ( - \eta )} \right] = 0,$$

here $C_{i} \left( {i = 1, \ldots ,5} \right)$ characterizes the arbitrary constants.

The final solutions $\left( {f_{m} ,\,\theta_{m} } \right)$ with the association of special solutions $\left( {f_{m}^{ * } \left( \eta \right),\,\theta_{m}^{ * } \left( \eta \right)} \right)$ are disclosed by

26 $$f_{m} \left( \eta \right) = f_{m}^{?} \left( \eta \right) + C_{1} + C_{2} \eta + C_{3} e^{ - \eta } ,$$

27 $$\theta_{m} \left( \eta \right) = \theta_{m}^{?} \left( \eta \right) + C_{4} e^{\eta } + C_{5} e^{ - \eta } ,$$

### Convergence analysis

**Figure 1:**
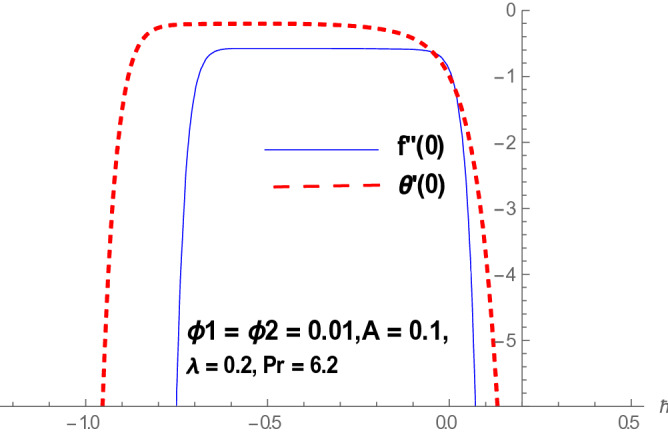
h-Curve for $f(\eta )$ and $\theta (\eta ).$

Homotopy analysis method is that method which gave us freedom to choose and control the convergence region. Figure 1 reflects the $\hbar$-curves for $f^{\prime}(\eta )$ and $\theta (\eta ).$ The ranges for $\hbar_{f}$ and $\hbar_{\theta }$ are $- 0.6 \le \hbar_{f} \le - 0.1$ and $- 0.8 \le \hbar_{\theta } \le - 0.5.$

## Discussion

**Figure 2:**
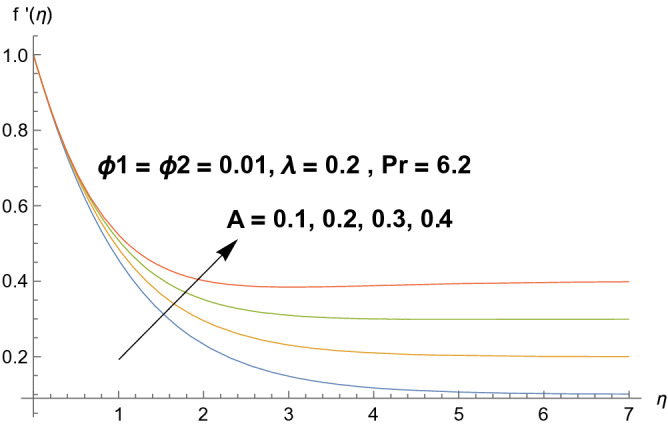
Illustration of A on $f^{\prime}(\eta ).$

**Figure 3:**
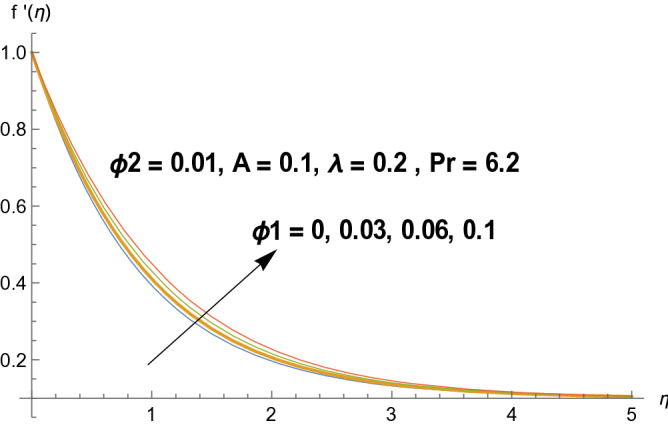
Illustration of $\Phi_{1}$ on $f^{\prime}(\eta ).$

**Figure 4:**
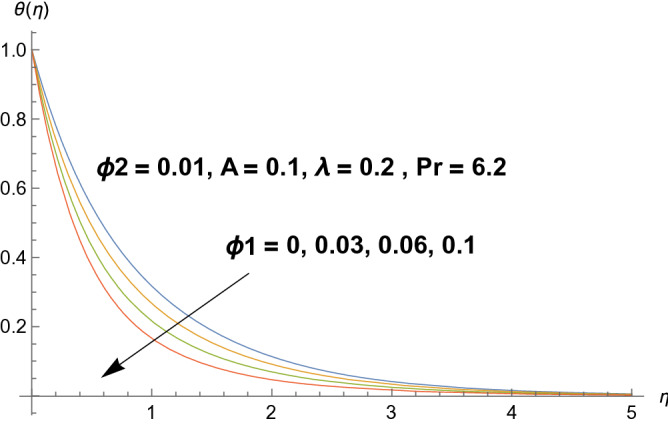
Illustration of $\Phi_{1}$ on $\theta (\eta ).$

**Figure 5:**
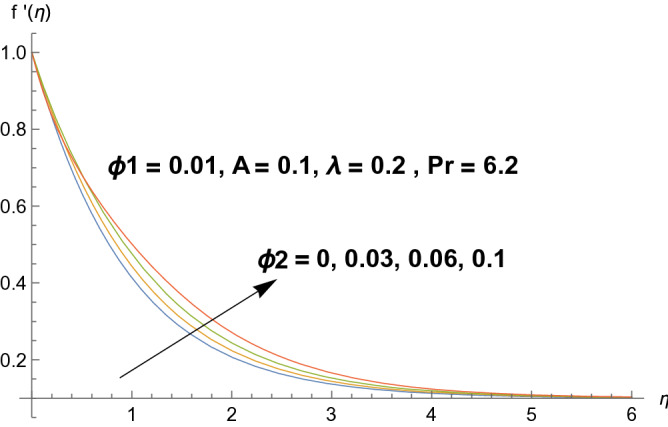
Illustration of $\Phi_{2}$ on $f^{\prime}(\eta ).$

**Figure 6:**
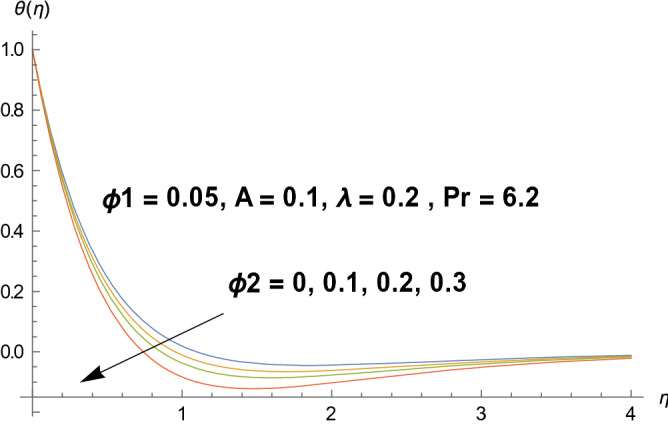
Illustration of $\Phi_{2}$ on $\theta (\eta ).$

**Figure 7:**
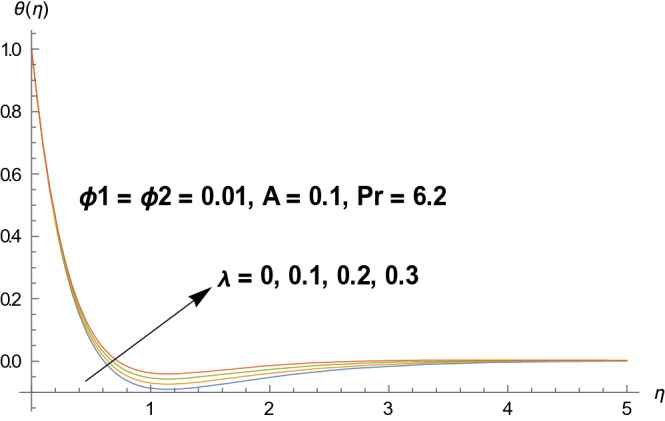
Illustration of $\lambda$ on $\theta (\eta ).$

Graphical demonstration of influential parameters for flow and heat transmission are given in this portion. Thus figures are portrayed. Effect of velocity ratio parameter $A$ on velocity profile is illustrated in Fig. 2. It is noticed that both velocity profile and momentum boundary layer thickness enhanced for increased velocity ratio parameter. Physically magnified ratio parameter prop up the free stream velocity which assists the velocity up gradation. Figure 3 is plotted for polystyrene particles volume fraction $\Phi_{1}$ versus velocity profile. It is observed that velocity profile grows for enhanced polystyrene particles volume fraction. This is because nano particle concentration enhances near the plate which inturns intensifies the velocity field. Influence of polystyrene particles volume fraction on temperature profile is observed in Fig. 4. Opposite behavior is detected for velocity and temperature distributions. Both temperature profile and thermal boundary layer thickness gets steeper. Actually nanoparticles disperse in the fluid and reduces the temperature. Figure 5 depicts the behavior of titanium oxide $\left( {{\text{TiO}}_{2} } \right)$ particles volume fraction $\Phi_{2}$ on velocity field. Titanium oxide nanoparticles strengthens the velocity field. Higher concentration of titanium oxide $\left( {{\text{TiO}}_{2} } \right)$ particles provides significant amount of particles near the plate consequently velocity increases. Figure 6 indicates the impact of titanium oxide $\left( {{\text{TiO}}_{2} } \right)$ particles volume fraction $\Phi_{2}$ on temperature distribution. Same trend is obtained as for velocity field. Nano particles volume fraction enhances the thermal conductivity of a base fluid. Therefore temperature distribution increases. Figure 7 indicates the influence of heat generation/absorption parameter $\lambda$ on temperature profile. Heat generation/absorption parameter $\lambda$ strengthens the thermal boundary layer and temperature profile. In the course of heat generation activity extra heat will be generated and eventually temperature distribution boost.

### Entropy production

**Figure 8:**
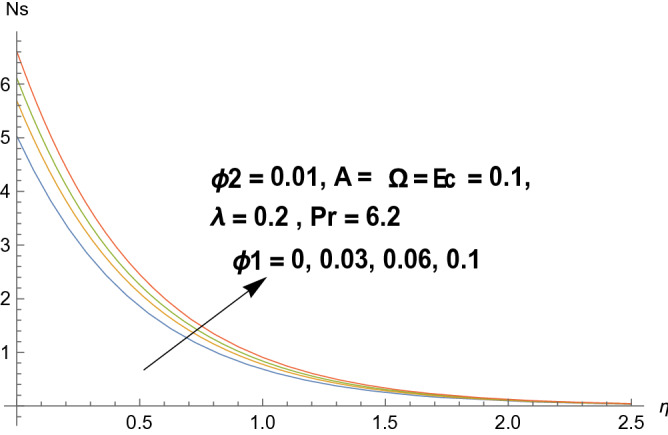
Illustration of $\Phi_{1}$ on $N_{s}$.

**Figure 9:**
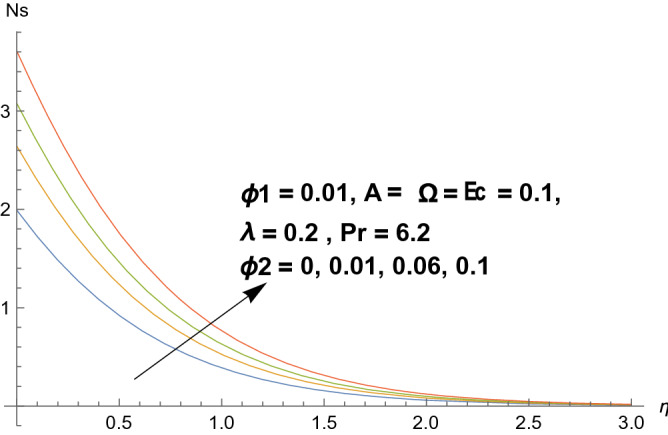
Illustration of $\Phi_{2}$ on $N_{s}$.

**Figure 10:**
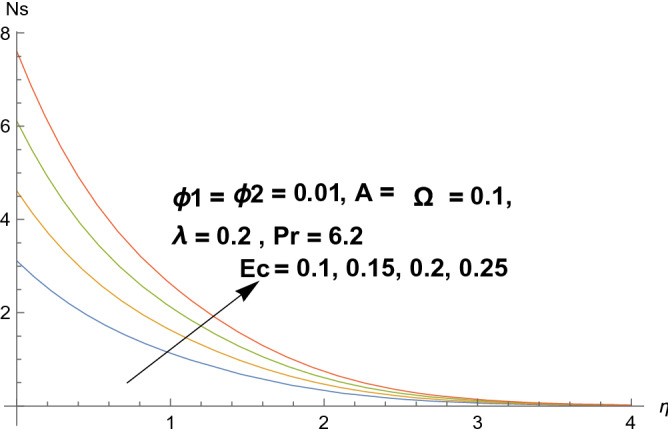
Illustration of $Ec$ on $N_{s}$.

**Figure 11:**
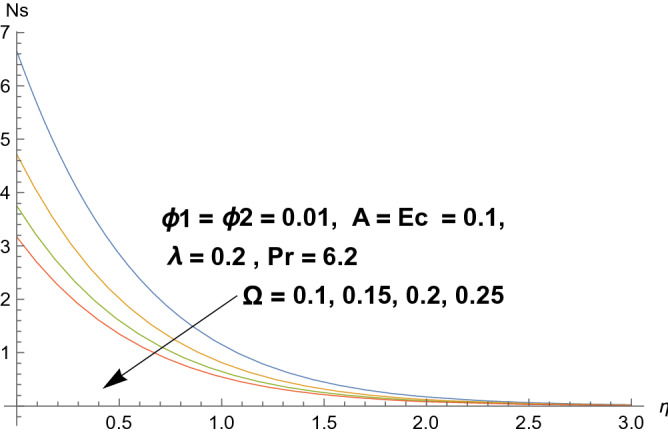
Illustration of $\Omega$ on $N_{s}$.

**Figure 12:**
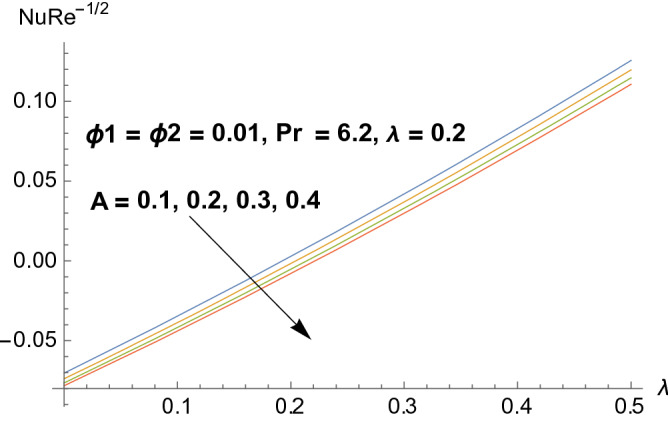
Illustration of A and $$\lambda$$ on $$Nu{\text{Re}}^{{{\raise0.7ex\hbox{${ - 1}$} \!\mathord{\left/ {\vphantom {{ - 1} 2}}\right.\kern-\nulldelimiterspace} \!\lower0.7ex\hbox{$2$}}}}$$.

**Figure 13:**
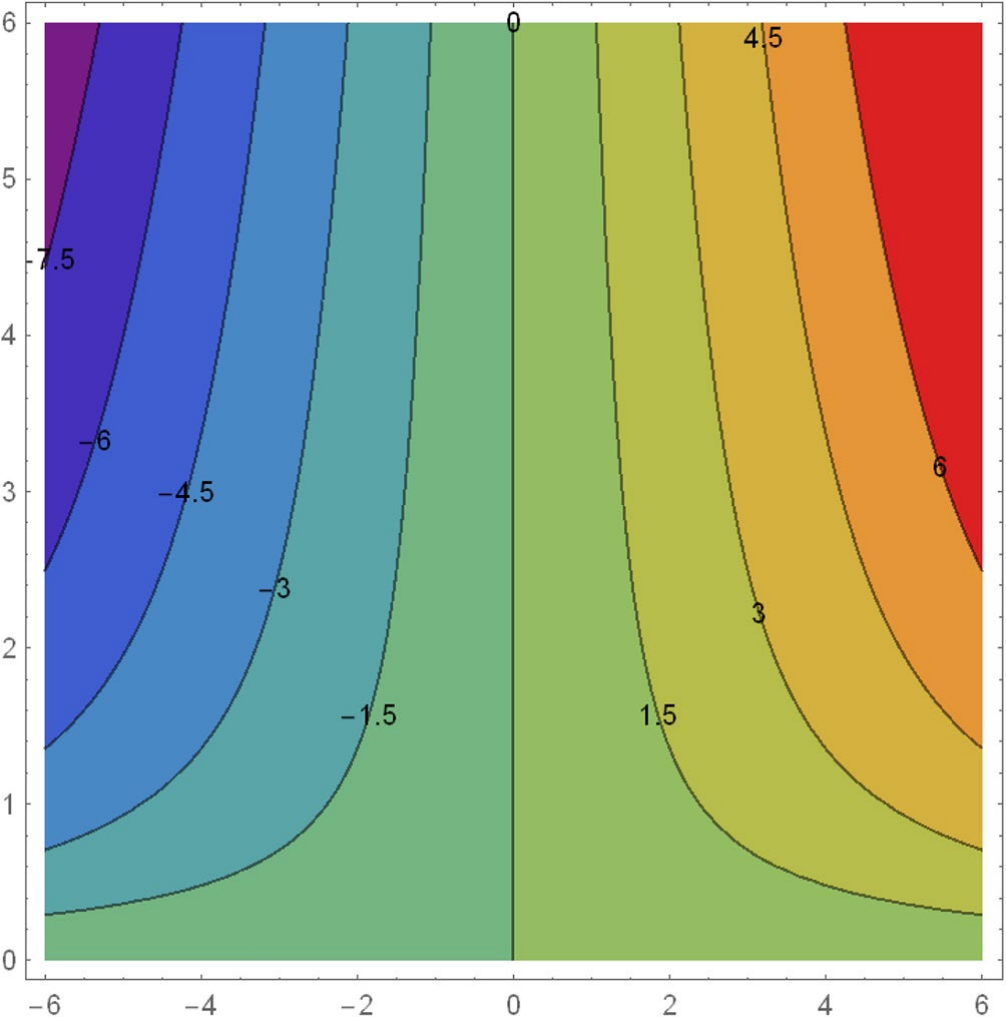
Flow pattern for streamlines.

Figures 8, 9, 10, and 11 reflects the influences of various parameters on entropy generation. Figures 8 and 9 represents the effect of polystyrene particles volume fraction $\Phi_{1}$ and titanium oxide particles volume fraction $\Phi_{2}$ on entropy generation. Results shows that both polystyrene particles volume fraction and titanium oxide particles volume fraction enhances the entropy generation rate. To determine the impact of Eckert number $Ec$ on entropy generation, Fig. 10 is sketched. It is noticed that entropy generation rate is increasing function of Eckert number. Actually due to enlarged Eckert number $Ec$ supplementary heat will be induced and therefore entropy generation dominates. Figure 11 portrayed the footprints of temperature difference parameter $\Omega$ on entropy production rate. Opposite behavior has been perceived as compared to Eckert number. Entropy generation rate diminishes when temperature difference parameter grows. Figure 12 reflects the influence of velocity ratio parameter $A$ and heat generation/absorption parameter $\lambda$ on heat transfer rate. It is noticed that heat transfer rate decays for increasing velocity ratio parameter $A$ and heat generation/absorption parameter $\lambda$. Actually heat generation/absorption parameter strengthens the temperature profile. Therefore, less heat will be transferred and heat transfer rate diminishes. Figure 13 demonstrates the streamlines behavior of flow.

Nomenclature for physical parameters and symbols are given in Table 1. Table 2 represents numerical values of thermophysical properties of Polystyrene and Titanium Oxide particles (nano particles) and Table 3 express the mathematical formulation of hybrid nanofluid and base fluid. Thermal conductivity of nanofluids was proposed by Hamilton and Crosser^57^. Here n is the empirical shape factor in order to account the effect of particles shape and can be varied from 0.5 to 6.0. The shape factor n is given by $\frac{3}{\psi }$, where $\psi$ is the particles sphericity, defined as surface area of a sphere to the surface area of the particle. Therefore for spherical nanoparticles n = 3. This case of Hamilton and Crossere model (n = 3) is the same as Maxwell model^58^. Similarly density,specific heat and dynamic viscosity of hybrid nanoparticles were used in literature^59–61^. Table 4 expresses the numerical values of skin friction coefficient for different physical parameters. It is observed that drag force decreases for both $\Phi_{1}$ (nano particle volume fraction of polystyrene) and $\Phi {}_{2}$ (nano particle volume fraction of titanium Oxide). Physically, increase in velocity profile educes the drag force for $\Phi_{1}$ and $\Phi_{2}$. Whereas opposite behavior is noticed for $A$ (velocity ratio parameter). Table 5 shows the numerical values of heat transfer rate for unlike physical parameters. Decay in rate of heat transfer is noticed for enlarged $\Phi_{1}$ (nano particle volume fraction of polystyrene) whereas increasing trend is observed for $\Phi_{2}$ (nano particle volume fraction of titanium Oxide) and $\lambda$ (heat generation/absorption parameter). Table 6 is prepared for the analysis of surface drag coefficient $(f^{\prime\prime}(0))$ with published works of Mahapatra and Gupta^62^, Pop et al.^63^, Sharma and Singh^64^ and Masood et al.^55^ in limiting case. Both results are in good manner.

**Table 1:** Nomenclature table for physical parameters and symbols^54^.

Nomenclature	
$k_{f}$	Thermal conductivity of water (Wm^−1^ K^−1^)
$k_{nf}$	Thermal conductivity of nanofluid (Wm^−1^ K^−1^)
$k_{hnf}$	Thermal conductivity of hybrid nanofluid (Wm^−1^ K^−1^)
$k_{s1}$	Thermal conductivity of polystyrene (Wm^−1^ K^−1^)
$k_{s2}$	Thermal conductivity of Titanium Oxide (Wm^−1^ K^−1^)
$a,b$	Dimensionless constants
$f^{\prime}$	Dimensionless velocity
$T$	Fluid temperature (K)
$T_{0}$	Reference temperature (K)
$T_{\infty }$	Ambient temperature (K)
$T_{w}$	Surface temperature (K)
$U_{w} (x)$	Stretching velocity (m s^−1^)
$U_{e} (x)$	Free stream velocity (m s^−1^)
$\Pr$	Prandtl number
$A$	Velocity ratio parameter
$\lambda$	Heat generation/absorption parameter
$Q$	Heat generation/absorption coefficient
$C_{f}$	Local skin friction coefficient
${\text{Re}}_{x}$	Local Reynold number
$Nu$	Nusselt number
$q_{w}$	Heat flux
**Greek symbols**	
$\rho_{f}$	Density of water (kg m^−3^)
$\rho_{nf}$	Density of nanofluid (kg m^−3^)
$\rho_{hnf}$	Density of hybrid nanofluid (kg m^−3^)
$\rho_{s1}$	Density of polystyrene (kg m^−3^)
$\rho_{s2}$	Density of titanium oxide (kg m^−3^)
$\mu_{f}$	Dynamic viscosity of water (kg m^−1^ s^−1^)
$\mu_{hnf}$	Dynamic viscosity of hybrid nanofluid (kg m^−1^ s^−1^)
$\alpha_{hnf}$	Thermal diffusivity of hybrid nanofluid (m^2^s^−1^)
$\upsilon_{hnf}$	Kinematic viscosity of hybrid nanofluid (m^2^s^−1^)
$\eta$	Similarity variable
$\Phi_{1}$	Nano particle volume concentration of polystyrene
$\Phi_{2}$	Nano particle volume concentration of TiO_2_
$(\rho C)_{f}$	Specific heat of water (J K^−1^ m^−3^)
$(\rho C)_{hnf}$	Specific heat of hybrid nanofluid (J K^−1^ m^−3^)
$(\rho C)_{s1}$	Specific heat of polystyrene (J K^−1^ m^−3^)
$(\rho C)_{s2}$	Specific heat of TiO_2_ (J K^−1^ m^−3^)
$\theta$	Dimensionless temperature
$\tau_{w}$	Wall shear stress (kg 3^−1^ s^−2^)

**Table 2:** Thermo physical properties of base fluid and nano particles^65,66^.

Base fluid/nano particles	*ρ* (kg m^−3^)	*C*_*p*_ (J K^−1^)	*k* (W K^−1^ m^−1^)
Water (H_2_O)	997.1	4179	0.613
Polystyrene	1053	1210	0.16
Titanium Oxide (TiO_2_)	4250	686.2	8.9538

**Table 3:** Mathematical formulation of thermo physical properties of hybrid nanofluid^67–70^.

Properties	Hybrid nanofluid
Dynamic viscocity (N s m^−2^)	$\mu_{hnf} = \frac{{\mu_{f} }}{{(1 - \Phi_{1} )^{2.5} (1 - \Phi_{2} )^{2.5} }}$
Density (kg m^−3^)	$\rho_{hnf} = [(1 - \Phi_{2} )\{ (1 - \Phi_{1} )\rho_{f} + \Phi_{1} \rho_{s1} \} + \Phi_{2} \rho_{s2} ]$
Thermal conductivity (W K^−1^ m^−1^)	$\frac{{k_{hnf} }}{{k_{nf} }} = \frac{{k_{s2} + (n - 1)k{}_{nf} - (n - 1)\Phi_{2} (k_{nf} - k_{s2} )}}{{k_{s2} + (n - 1)k_{nf} + \Phi_{2} (k_{nf} - k_{s2} )}}$ where $\frac{{k_{nf} }}{{k_{f} }} = \frac{{k_{s1} + (n - 1)k_{f} - (n - 1)\Phi {}_{1}(k_{f} - k_{s1} )}}{{k_{s1} + (n - 1)k_{f} + \Phi_{1} (k_{f} - k_{s1} )}}$
Heat capacity (J K^−1^)	$[(1 - \Phi_{2} )\{ (1 - \Phi_{1} )(\rho C_{p} )_{f} + \Phi_{1} (\rho C_{p} )_{s1} \} + \Phi_{2} (\rho C_{p} )_{s2} ]$

**Table 4:** Skin friction coefficient for different parameters when $\lambda = 0.1,Pr = 6.2.$

$\Phi_{1}$	$\Phi_{2}$	$A$	$f^{\prime\prime}(0)$
0.01	0.01	0.1	0.0042989
0.02			0.0037307
0.03			0.0031260
0.01			0.0042989
	0.02		0.0040934
	0.03		0.003915
	0.01		0.0042989
		0.2	0.0073951
		0.3	0.013953

**Table 5:** Nusselt number for different parameters when $A = 0.1,\Pr = 6.2.$

$\Phi_{1}$	$\Phi_{2}$	$\lambda$	$- \theta^{\prime}(0)$
0.01	0.01	0.1	0.015735
0.02			0.015511
0.03			0.015359
0.01			0.015735
	0.02		0.016164
	0.03		0.016529
	0.01		0.015735
		0.2	0.015838
		0.3	0.016084

**Table 6:** Review of skin friction coefficient $(f^{\prime \prime } (0))$ with previous published work of Mahapatra and Gupta^62^, Pop et al.^63^, Sharma and Singh^64^ and Masood et al.^55^ for different values of $A$ when $\Phi_{1} = \Phi_{2} = 0.$

A	Mahapatra and Gupta^62^	Pop et al.^63^	Sharma and Singh^64^	Masood et al.^55^	Present results
0.1	− 0.9694	− 0.9694	− 0.969483	− 0.96939	− 0.96939
0.2	− 0.9181	− 0.9181	− 0.9181069	− 0.91811	− 0.91811
0.5	− 0.6673	− 0.6673	− 0.667263	− 0.66726	− 0.66726

## Conclusions

Influence of heat generation/absorption and stagnation point on hybrid nanofluid are taken into account. Hybrid nanofluid contains Polystyrene and TiO_2_ nanoparticles with water as a transiet fluid. The disruptive results for the sophisticated parameters are displayed graphically. The majour outcomes are as persued.

- Volume fraction of polystyrene particles $\Phi_{1}$ enlarged the velocity profile and degrade the temperature profile.
- Volume fraction of titanium oxide $\Phi_{2}$ particles magnifies the velocity field.
- Velocity ratio parameter enhanced the velocity field.
- Heat generation/absosption parameter emlarged the temperature profile.
- Entropy generation intensifies for both polystyrene and titanium oxide parcticles.
- Eckert number $Ec$ also amplifies the entropy generation strength.
- Temperature difference parameter decays the entropy generation.

The preference is that the contemporaneous analysis will be extremely beneficial for modeling better flow obstacles specifically in biomedical industry, for cancer treatment, aerodynamic industry, power generation, nuclear reactors and solar thermal absorbers.
